# Frozen blood clots can be used for the diagnosis of distinct *Plasmodium* species in man and non-human primates from the Brazilian Atlantic Forest

**DOI:** 10.1186/s12936-018-2485-0

**Published:** 2018-09-24

**Authors:** Filipe Vieira Santos de Abreu, Larissa Rodrigues Gomes, Aline Rosa Lavigne Mello, Cesare Bianco-Júnior, Anielle de Pina-Costa, Edmilson dos Santos, Danilo Simonini Teixeira, Patrícia Brasil, Cláudio Tadeu Daniel-Ribeiro, Ricardo Lourenço-de-Oliveira, Maria de Fátima Ferreira-da-Cruz

**Affiliations:** 1 Laboratório de Mosquitos Transmissores de Hematozoários, Instituto Oswaldo Cruz, Fiocruz, Rio de Janeiro, Brazil; 2 Instituto Federal do Norte de Minas Gerais Campus Salinas, Minas Gerais, Brazil; 3 Laboratório de Pesquisa em Malária, Instituto Oswaldo Cruz, Fiocruz, Rio de Janeiro, Brazil; 4 Centro de Pesquisa, Diagnóstico e Treinamento em Malária, Instituto Oswaldo Cruz, Fiocruz, Rio de Janeiro, Brazil; 5 Laboratório de Doenças Febris Agudas, Instituto Nacional de Infectologia Evandro Chagas, Fiocruz, Rio de Janeiro, Brazil; 6 Divisão de Vigilância Ambiental em Saúde, Secretaria de Saúde do Rio Grande do Sul, Porto Alegre, Rio Grande do Sul Brazil; 7 Faculdade de Agronomia e Veterinárias, Universidade de Brasília, Brasília, Brazil

**Keywords:** Blood clot, *Plasmodium*, *Alouatta guariba clamitans*, Non-human-primates, Yellow fever, Atlantic Forest

## Abstract

**Background:**

Zoonotic infections with epidemic potential, as non-human primate malaria and yellow fever (YF), can overlap geographically. Optimizing a small blood sample for diagnosis and surveillance is of great importance. Blood are routinely collected for YF diagnosis and blood clots usually discarded after serum obtention. Aiming to take sample advantage, the sensitivity of a PCR using extracted DNA from long-term frozen clots from human and non-human primates for detection of *Plasmodium* spp. in low parasitaemia conditions was assayed.

**Results:**

Malaria diagnosis with DNA extracted from blood clots generated results in agreement with samples obtained with whole blood, including mixed *Plasmodium vivax/simium* and *Plasmodium malariae/brasilianum* infections.

**Conclusion:**

Blood clots from human and non-human primates may be an important and low cost source of DNA for malaria surveillance in the Atlantic Forest.

## Background

Some zoonotic infections with epidemic potential may overlap geographically. Their surveillance and follow-up during epizooties and epidemics depends upon the screening for distinct infectious agents often in a single blood sampling from animal reservoirs. Frequently, reservoirs are low weight animals, and sometimes, hypovolaemia at the sampling moment prevents the collection of the ideal amount of blood. Therefore, optimizing the blood sample may be critical for diagnosis of different infections at the time.

Non-human primates (NHPs) are reservoirs of main zoonotic agents, such as *Plasmodium* spp. and yellow fever virus (YFV). For instance, in South America, the YFV coexists in the same areas where *Plasmodium* spp. from NHP origin has been recorded for decades [1–3]. Thus, simultaneous surveillance of these zoonotic infections in a single NHP blood sampling is very important.

Recently, outbreaks of human malaria and YF acquired in the sylvatic transmission cycle have been reported in the Atlantic Forest localities in Southeast Brazil, the states of Espírito Santo (ES) and Rio de Janeiro (RJ) were the most affected by both outbreaks [4–8]. Indeed, the most severe yellow fever outbreak in the last seven decades reemerged in the Atlantic Forest areas with 689 humans and 1394 NHPs confirmed deaths [7, 9]. In the same way, outbreaks caused by *Plasmodium simium*, a NHP parasite closely related to *Plasmodium vivax*, have been recently reported [1, 5, 10–14], as well as outbreaks by *Plasmodium brasilianum*, another New World NHP malaria parasite morphologically indistinguishable to *Plasmodium malariae* [1]. Conventional PCR protocols for malaria diagnosis are adopted worldwide [e.g. 15, 16] and also do not distinguish *P. vivax* and *P. malariae* from *P. simium* and *P. brasilianum*, respectively [5, 12, 17]. It has been postulated that *P. vivax* can only be differentiated from *P. simium* by two specific Single Nucleotide Polymorphisms (SNPs) in mitochondrial DNA from human or NHP red blood cells or whole blood [5, 6, 18]. *Plasmodium simium* parasitaemia is usually low to scanty both in NHP and human hosts. Concerning YFV, the Brazilian Ministry of Health preconizes the active surveillance of NHPs, which requires their capture with traps or anesthetic darts together with blood sampling for serological and molecular assays. Thus, blood is regularly collected in tubes without anticoagulant to obtain serum [19], normally the blood clot discarded after serum collection.

The main host of both *P. simium* and the YFV in Southeastern and Southern Brazil are howler monkeys, genus *Alouatta* [1, 19]. These animals hardly ever enter traps and their capture in the forest canopy is difficult and expensive. Due to the difficulties to obtain blood samples from wild howler monkeys and other neotropical NHPs, it is important to optimize such samples. Therefore, the methodology proposed by Lundblom et al. [20] was improved and assayed for the detection of *Plasmodium* spp. DNA in blood clots from man and NHPs taking into account the low parasitemia conditions frequently associated with these malaria infections.

## Methods

### Non-human primate samples

During a YFV surveillance in 2016, two *Alouatta guariba clamitans* (M1 and M3) and one *Callithrix* hybrid (M2) (Table 1) were captured in the Atlantic Forest areas of Rio de Janeiro state, Brazil, under the technical guidelines of sampling and security measures [19]. A total of 5 and 3 mL of venal blood was, respectively, collected from femoral puncture of M1/M3 and M2 and then equally split into two tubes, one without anticoagulant and the other containing EDTA. Following centrifugation (2000*g* × 10 min), sera, clot, plasma and whole blood samples were stored at − 80 °C until DNA extraction. Whole blood and clot samples were not depleted of leucocytes and platelets.

**Table 1:** PCR results from whole blood and blood clots according to malaria species and estimated parasitaemia according to microscopy examination (thick and thin blood film)

Code	Species	Parasitaemia (parasites/μL)	PCR^a^: whole blood	PCR^a^: 1st blood clot DNA extraction^b^	PCR^a^: 2nd blood clot DNA extraction^b^	PCR^a^: 3rd blood clot DNA extraction^b^
H1	*Homo sapiens*	208	*Plasmodium; P. vivax/P. simium*	*Plasmodium; P. vivax/P. simium*	*Plasmodium; P. vivax/P. simium*	*Plasmodium*; *P. vivax/P. simium*
H2	*Homo sapiens*	368	*Plasmodium; P. vivax/P. simium*	*Plasmodium; P. vivax/P. simium*	*Plasmodium; P. vivax/P. simium*	*Plasmodium; P. vivax/P. simium*
H3	*Homo sapiens*	0	−	−	−	−
M1	*Alouatta g. clamitans*	300	*Plasmodium; P. vivax/P. simium* and *P. malariae/P. brasilianum*	*Plasmodium; P. vivax/P. simium* and *P. malariae/P. brasilianum*	*Plasmodium; P. vivax/P. simium and P. malariae/P. brasilianum*	*Plasmodium; P. vivax/P. simium and P. malariae/P. brasilianum*
M2	*Callithrix* hybrid	0	−	−	−	−
M3	*Alouatta g. clamitans*	40	*P. malariae/P. brasilianum*	*P. malariae/P. brasilianum*	*P. malariae/P. brasilianum*	*P. malariae/P. brasilianum*

^a^Gene targets were cysteine proteinase for *P. vivax*, ssrRNA for *P. malariae* and 18S rRNA for genus *Plasmodium*; ^b^ DNA extractions from clots disrupted by high-speed shaking (6000 rpm/40 s) in lysis buffer, without glass beads; −: negative result

### Human samples

Five mL blood samples collected in January 2017 from *P. vivax* patients (H1 and H2) infected in the Atlantic Forest areas of Rio de Janeiro state, Brazil and from a clinically healthy donor (H3) were used. Both whole blood and blood clots were obtained, processed and stored as described for NHP samples.

### Parasitological diagnosis

Giemsa-stained thick and thin blood films of human and NHP samples were examined prior to PCR assays. Samples H1, H2, M1 and M3 were positive, displaying 208, 368, 300 and 40 malaria parasites/µL, respectively. The remaining two samples, a human (H3) and one NHP (M2) were negative (Table 1).

### DNA extraction and PCR

After thawing, 600–800 μL of lysis buffer (AL lysis buffer provided in QIamp^®^ DNA mini kit, Qiagen) were added to 300–400 μL (2:1) of each blood clot in tubes containing or not glass beads, a high-speed shaking (6000 rpm/40 s; Bertin Precellys 24) was performed for clot disruption. Afterwards, this DNA was extracted with the QIAamp^®^ DNA mini kit, according to manufacturer’s instructions, except for the elution step volume that was made in 50 instead of 100 μL. The unprocessed whole blood DNA was extracted from around 600 μL with both the same kit and elution procedures. In order to check DNA extraction efficiency, 3 sets of extractions were realized with the same blood clot sample, as already described. The presence of gDNA was evaluated after electrophoresis (110 A/1 h) on ethidium bromide agarose gel under UV light. To check PCR assay precision, these samples were tested in three replicates (intra-assays) on each of three different days (between assays).

For the conventional PCR assays, all DNA samples were tested for 18 s rRNA *Plasmodium* genus-specific gene [21], and than for cysteine proteinase *P. vivax* [22] and ssrRNA *P. malariae* genes [15]. The sensitivity threshold of the genus [21] and *P. vivax* assay [22] specific protocols optimized and routinely used are 0.5 and 0.019 parasites/μL respectively, while that of the *P. malariae* PCR is of 1 parasite/μL according to the authors [15]. PCR products were visualized under UV light after electrophoresis on 2% agarose gels. DNA from patients infected by *P. vivax* or *P. malariae* was the positive control in each round of amplification. Non-infected human and NHP DNA as well as blank samples (no DNA) were the negative controls.

### Sensitivity comparison assay

In order to compare the sensitivity of PCR based on blood clots we used similar quantities of blood cells from whole blood and blood clot samples from another *A. g. clamitans* (M3) naturally infected with *P. malariae* (parasitaemia of 120 parasites/µL). Blood clots are composed of red and white blood cells, platelets and fibrin, which together roughly correspond to almost 50% of all human and NHP blood volume. Thus, aiming to equalize the amount of blood cells we used 300 μL and 600 μL of clot and whole blood, respectively. Whole blood and disrupted blood clot samples were diluted (1:2, 1:4, 1:10; 1:100; 1:1000; 1:10,000, 1:100,000) (Table 2) and DNA extractions from each dilution were processed for *P. malariae* PCR amplification, as described above [15]. In addition, we performed PCRs assays changing the last hold cycle of the first and second rounds to 58°–5 min instead of 58°–2 min and 72°–10 min instead of 72°–5 min as well as the number of cycles in the second round changed to 35 instead of 30.

**Table 2:** PCR results from different *Plasmodium malariae* concentrations of blood clot and whole blood samples from an infected *Alouatta g. clamitans* (M3) monkey

M3 *Alouatta g. clamitans*		Dilutions							
Conditions	Sample	1	1:2	1:4	1:10	1:100	1:1000	1:10,000	1:1,000,000
Regular PCR^a^ conditions	Blood clot^b^	*+*	*+*	*+*	*+*	*−*	*−*	*−*	*−*
	Whole blood	*+*	*+*	*+*	*+*	*+*	*−*	*−*	*−*
Improved PCR^a^ conditions	Blood clot^b^	+	+	+	+	+	*−*	*−*	*−*
	Whole blood	+	+	+	+	+	+	*−*	*−*

^a^The gene target was ssrRNA; ^b^ DNA extractions from clots disrupted by high-speed shaking (6000 rpm/40 s) in lysis buffer, without glass beads; + and −: positive and negative result, respectively

## Results

The PCR results with DNA extracted from clots were in agreement with those of the whole blood (Table 1). Concerning extraction without beads, there were small residual fragments, but they were dissolved in subsequent incubations, the gDNA strongly detectable under UV light. Thus, the DNA clot PCR results reported herein were from disruptions performed without glass beads. H1 and H2 samples were PCR positive for genus *Plasmodium* and *P. vivax* and negative for *P. malariae,* whereas M1 (*A. g. clamitans*) was positive for genus, *P. vivax* and *P. malariae*. Despite the low parasitaemia, samples were consistently intra-assay (repeatability) and between assay (reproducibility) positive on the 3 different days (Table 1). Inversely, clot disruption with glass beads, besides requiring centrifugation steps, generated a weak and indistinct gDNA band under UV light, PCR reactions negative regardless of the PCR target.

Concerning sensitivity*, P. malariae* was detected in blood clot and whole blood of M3 till the dilution of 1:10 and 1:100 that corresponds to 4 and 0.4 parasites/µL, respectively. By increasing the number of cycles of the PCR, the limit of detection (LoD) increased to 0.4 parasites/µL for blood clot samples and to 0.04 parasites/µL for whole blood samples (Table 2).

## Discussion

Human and non-human primate blood clots could be important sources of DNA that can be advantageous for *Plasmodium* spp. detection even in low parasitaemias. This is the first report of blood clots for the detection of *Plasmodium* DNA in NHPs as well as an unprecedented description of *P. vivax/simium* and *P. malariae*/*brasilianum* detection in clot samples even with low parasitaemias. In fact, blood clot disruption with high speed shaking was only reported for the detection of *P. falciparum* in larger volume samples of human blood clots with no information of a precision (repeatability and reproducibility) parameter [20]. It was also demonstrated that the LoD from the clot was smaller than that of whole blood under standard PCR conditions. However, when the number of cycles was changed, the sensitivity increased for both clot and whole blood samples. Thus, increasing the number of cycles can compensate a possible loss of sensitivity for blood clots with very low parasitaemia. Therefore, the use of often-discarded blood clots allows the investigation of malaria parasites in the same blood sampling from which serum was extracted for simultaneous surveillance of YFV as well as other arboviruses in NHPs.

Interestingly, the high-speed shaking technique without the use of beads exhibited best blood clot disruption performance. This corresponds to an advantage by reducing risk of contaminations due to handling as described for other disruption techniques [23–25]. In addition to malaria diagnosis, PCR templates from NHP blood can be used for epidemiological, ecological and evolutionary purposes. In fact, NHP trypanosomes infecting man, such as *Trypanosoma cruzi*, can be even more sensitively detected in blood clots when compared to whole blood and leukocyte samples [26] or serum in the case of detecting fungi causing aspergillosis in man [27].

Furthermore, human blood clots stored at − 20 °C from 1 to 2.5 years were still useful for the investigation of single-nucleotide polymorphisms [28]. Indeed, PCR results with blood clots were essentially equivalent to whole blood (Tables 1, 2), as demonstrated in this paper with blood clots stored at − 80 °C for around 2 years, revealing the maintenance quality of the DNA template. Consequently, blood clot storage may be also a stable source of primate genetic samples for several phylogeny, dispersion and gene flow studies as whole blood tissues, hair or even feces, which have already been adopted as DNA sources by several researchers [29–33] (non-exhaustive list). Along with increasing deforestation, studies of habitat fragmentation effects, especially in small populations of endangered NHPs, are necessary to support management strategies [34], and clotted blood could also be a stable genetic bank for several populations.

Finally in view of the results reported here, we suggest that the Brazilian Ministry of Health surveillance protocols recommend NHP blood clot collection storage and examination. This would indeed correspond to a low-cost initiative that could optimize the results of NHP capture and safety as well as contribute to knowledge expansion in different fields of primatology and epidemiology for the protection of man against zoonotic diseases.

## Conclusion

In conclusion, blood clots can be a valuable source of genetic information, optimizing costs, handling time and reducing the amount of blood collected from each subject. Thereby, the creation of a blood clot bank could aid the resolution of several ecological, evolutionary and epidemiological issues.
