# Correction to: Frozen blood clots can be used for the diagnosis of distinct *Plasmodium* species in man and non-human primates from the Brazilian Atlantic Forest

**DOI:** 10.1186/s12936-019-2804-0

**Published:** 2019-05-14

**Authors:** Filipe Vieira Santos de Abreu, Larissa Rodrigues Gomes, Aline Rosa Lavigne Mello, Cesare Bianco-Júnior, Anielle de Pina-Costa, Edmilson dos Santos, Danilo Simonini Teixeira, Patrícia Brasil, Cláudio Tadeu Daniel-Ribeiro, Ricardo Lourenço-de-Oliveira, Maria de Fátima Ferreira-da-Cruz

**Affiliations:** 1 Laboratório de Mosquitos Transmissores de Hematozoários, Instituto Oswaldo Cruz, Fiocruz, Rio de Janeiro, Brazil; 2 Instituto Federal do Norte de Minas Gerais Campus Salinas, Minas Gerais, Brazil; 3 Laboratório de Pesquisa em Malária, Instituto Oswaldo Cruz, Fiocruz, Rio de Janeiro, Brazil; 4 Centro de Pesquisa, Diagnóstico e Treinamento em Malária, Instituto Oswaldo Cruz, Fiocruz, Rio de Janeiro, Brazil; 5 Laboratório de Doenças Febris Agudas, Instituto Nacional de Infectologia Evandro Chagas, Fiocruz, Rio de Janeiro, Brazil; 6 Divisão de Vigilância Ambiental em Saúde, Secretaria de Saúde do Rio Grande do Sul, Porto Alegre, Rio Grande do Sul Brazil; 7 Faculdade de Agronomia e Veterinárias, Universidade de Brasília, Brasília, Brazil; 8 Centro Universitário Serra dos Órgãos (UNIFESO), Teresópolis, RJ 25964-004 Brazil

## Correction to: Malar J (2018) 17:338 10.1186/s12936-018-2485-0

Following publication of the original article [1], it was flagged that one of the authors (Anielle de Pina Costa) is missing an affiliation in the article.

Please note the author Anielle de Pina Costa also has this affiliation:

Centro Universitário Serra dos Órgãos (UNIFESO), Teresópolis/RJ, 25964-004, Brazil.

The author apologizes for this error.
